# Student satisfaction in the Russell Group and Non-Russell Group Universities in UK

**DOI:** 10.1016/j.dib.2018.11.124

**Published:** 2018-11-30

**Authors:** Mohammad Nurunnabi, Abdelhakim Abdelhadi

**Affiliations:** a Prince Sultan University, Rafha Street, PO Box – 66833, Riyadh 11586, Saudi Arabia; b St Antony’s College, University of Oxford, 62 Woodstock Road, Oxford OX2 6JF, UK

**Keywords:** Student satisfaction, National Student Survey (NSS), Russell Group, United Kingdom

## Abstract

Undeniably, student satisfaction in higher education is one of the important key factors for university ranking and league table. Accordingly, this article describes the student satisfaction data between Russell Group and Non-Russell Group universities in United Kingdom (UK). The data includes 19 Russell Group and 102 Non-Russell Group universities. We present some descriptive statistics of the variables included in the dataset. The results of the article are based on the two-Sample t-Test and CI. The findings from the data suggest that there is no statistically significant difference on student satisfaction rating between Russell Group and Non-Russell Group universities. This study has policy implications for the higher education in the UK.

**Specifications table**

**Table:** 

Subject area	Social sciences
More specific subject area	Education
Type of data	Tables and Figures
How data was acquired	Survey (The Higher Education Funding Council for England - Hefce, national database)
Data format	Raw, analyzed
Experimental factors	Student Satisfaction
Experimental features	Comparing Student Satisfaction between Russell Group Vs Non-Russell Group Universities
Data source location	121 Universities in United Kingdom
Data accessibility	Data are included in this article

## Value of the data

• There is lack of research on student satisfaction in the UK and hence this article aimed to find out the correlation of student satisfaction between Russell Group Vs Non-Russell Group Universities.
• The data were collected from the Higher Education Funding Council for England (Hefce), UK.
• The data can be used to identify the key performance factors associated with UK universities.
• The article could be useful for further study on the gender voices (student satisfaction) in UK and comparison with other countries.

The data did not present the respondents identity and hence confidentiality and anonymity of the respondents were maintained. The dataset provides an original contribution to the understanding of the student satisfaction in the UK.

## 1 Data

Student perception survey is the most common approach to evaluate the quality of universities [1], [2], [3], [4]. This is for quality improvement, in response to the increased international competition for students and operational funds in higher education [5], [6], [7].

Table 1 presents the sample of the study (n = 121). The study includes 19 Russell Group and 102 Non- Russell Group universities. Importantly, five renowned Russell Group universities were excluded due to the data availability: Cardiff University, Queen׳s University Belfast, University of Edinburgh, University of Glasgow, and University of Southampton.

**Table 1:** Sample of the study (n = 121).

**Sl**	**Institution**	**Russell Group**	**Non-Russell Group**	**UK Provider Ref. No.**
1	Imperial College London	Y		10003270
2	King׳s College London	Y		10003645
3	Queen Mary University of London	Y		10007775
4	The London School of Economics and Political Science	Y		10004063
5	The University of Birmingham	Y		10006840
6	The University of Leeds	Y		10007795
7	The University of Liverpool	Y		10006842
8	The University of Manchester	Y		10007798
9	The University of Nottingham	Y		10007154
10	The University of Sheffield	Y		10007157
11	The University of Warwick	Y		10007163
12	University College London	Y		10007784
13	University of Bristol	Y		10007786
14	University of Cambridge	Y		10007788
15	Durham University	Y		10007143
16	University of Exeter	Y		10007792
17	Newcastle University	Y		10007799
18	University of Oxford	Y		10007774
19	University of York	Y		10007167
20	Anglia Ruskin University		Y	10000291
21	Aston University		Y	10007759
22	Bath Spa University		Y	10000571
23	Birkbeck College		Y	10007760
24	Birmingham City University		Y	10007140
25	Bishop Grosseteste University		Y	10007811
26	Bournemouth University		Y	10000824
27	Brunel University London		Y	10000961
28	Buckinghamshire New University		Y	10000975
29	Canterbury Christ Church University		Y	10001143
30	City, University of London		Y	10001478
31	Courtauld Institute of Art		Y	10007761
32	Coventry University		Y	10001726
33	De Montfort University		Y	10001883
34	Edge Hill University		Y	10007823
35	Falmouth University		Y	10008640
36	Goldsmiths׳ College		Y	10002718
37	Guildhall School of Music & Drama		Y	10007825
38	Harper Adams University		Y	10040812
39	Heythrop College		Y	10007765
40	Kingston University		Y	10003678
41	Leeds Beckett University		Y	10003861
42	Leeds College of Art		Y	10003854
43	Leeds Trinity University		Y	10003863
44	Liverpool Hope University		Y	10003956
45	Liverpool John Moores University		Y	10003957
46	London Metropolitan University		Y	10004048
47	London South Bank University		Y	10004078
48	Loughborough University		Y	10004113
49	Manchester Metropolitan University		Y	10004180
50	Middlesex University		Y	10004351
51	Newman University		Y	10007832
52	Norwich University of the Arts		Y	10004775
53	Nottingham Trent University		Y	10004797
54	Oxford Brookes University		Y	10004930
55	Plymouth College of Art		Y	10005127
56	Ravensbourne		Y	10005389
57	Roehampton University		Y	10007776
58	Rose Bruford College of Theatre and Performance		Y	10005523
59	Royal College of Music		Y	10007778
60	Royal Holloway, University of London		Y	10005553
61	Royal Northern College of Music		Y	10007837
62	Sheffield Hallam University		Y	10005790
63	Southampton Solent University		Y	10006022
64	St Mary׳s University, Twickenham		Y	10007843
65	St. George׳s, University of London		Y	10007782
66	Staffordshire University		Y	10006299
67	Teesside University		Y	10007161
68	The Arts University Bournemouth		Y	10000385
69	The Conservatoire for Dance and Drama		Y	10001653
70	The Liverpool Institute for Performing Arts		Y	10003945
71	The Open University		Y	10007773
72	The Royal Academy of Music		Y	10007835
73	The Royal Agricultural University		Y	10005545
74	The Royal Central School of Speech and Drama		Y	10007816
75	The Royal Veterinary College		Y	10007779
76	The School of Oriental and African Studies		Y	10007780
77	The University of Bath		Y	10007850
78	The University of Bolton		Y	10006841
79	The University of Bradford		Y	10007785
80	The University of Chichester		Y	10007137
81	The University of Cumbria		Y	10007842
82	The University of East Anglia		Y	10007789
83	The University of Essex		Y	10007791
84	The University of Huddersfield		Y	10007148
85	The University of Hull		Y	10007149
86	The University of Kent		Y	10007150
87	The University of Lancaster		Y	10007768
88	The University of Leicester		Y	10007796
89	The University of Northampton		Y	10007138
90	The University of Reading		Y	10007802
91	The University of Salford		Y	10007156
92	The University of Surrey		Y	10007160
93	The University of West London		Y	10006566
94	The University of Westminster		Y	10007165
95	The University of Wolverhampton		Y	10007166
96	Trinity Laban Conservatoire of Music and Dance		Y	10008017
97	University College Birmingham		Y	10000712
98	University for the Creative Arts		Y	10006427
99	University of Bedfordshire		Y	10007152
100	University of Brighton		Y	10000886
101	University of Central Lancashire		Y	10007141
102	University of Chester		Y	10007848
103	University of Derby		Y	10007851
104	University of East London		Y	10007144
105	University of Gloucestershire		Y	10007145
106	University of Greenwich		Y	10007146
107	University of Hertfordshire		Y	10007147
108	University of Keele		Y	10007767
109	University of Lincoln		Y	10007151
110	University of Northumbria at Newcastle		Y	10001282
111	University of Plymouth		Y	10007801
112	University of Portsmouth		Y	10007155
113	University of St Mark & St John		Y	10037449
114	University of Sunderland		Y	10007159
115	University of Sussex		Y	10007806
116	University of the Arts, London		Y	10007162
117	University of the West of England, Bristol		Y	10007164
118	University of Winchester		Y	10003614
119	University of Worcester		Y	10007139
120	Writtle University College		Y	10007657
121	York St John University		Y	10007713

Note: Yes

National Student Survey (NSS) is compulsory for final year students on all courses in all higher education institutions in UK. Students respond based on a Likert scale where: 5 - Definitely agree, 4 - Mostly agree, 3 - Neither agree nor disagree, 2 - Mostly disagree, 1 - Definitely disagree. The following areas are covered in the following seven areas. In addition, the students respond to the Students’ Union question:

A. Teaching and learning

1. Staff are good at explaining things
2. Staff have made the subject interesting
3. Staff are enthusiastic about what they are teaching
4. The course is intellectually stimulating

A. Assessment and feedback

5. The criteria used in marking have been made clear in advance
6. Assessment arrangements and marking have been fair
7. Feedback on my work has been prompt
8. I have received detailed comments on my work
9. Feedback on my work has helped me clarify things I did not understand

A. Academic support

10. I have received sufficient advice and support with my studies
11. I have been able to contact staff when I needed to
12. Good advice was available when I needed to make study choices

A. Organization and management

13. The timetable works efficiently as far as my activities are concerned
14. Any changes in the course or teaching have been communicated effectively
15. The course is well organized and is running smoothly

A. Learning resources

16. The library resources and services are good enough for my needs
17. I have been able to access general IT resources when I needed to
18. I have been able to access specialized equipment, facilities or rooms when I needed to

A. Personal development

19. The course has helped me present myself with confidence
20. My communication skills have improved
21. As a result of the course, I feel confident in tackling unfamiliar problems

A. Overall satisfaction

22. Overall I am satisfied with the quality of the course

## 2 Experimental design, materials and methods

According to the Russell Group, the universities have significant social, economic and cultural impacts nationally and around the globe (https://russellgroup.ac.uk/about/our-universities). For example

• Russell Group universities produce more than two-thirds of the world-leading research produced in UK universities
• These universities support more than 300,000 jobs in UK with economic output is more than £32 billion every year.
• There were 417,000 undergraduates and 192,500 postgraduates were studying at a Russell Group university in 2015–16.

**Fig. 1:**
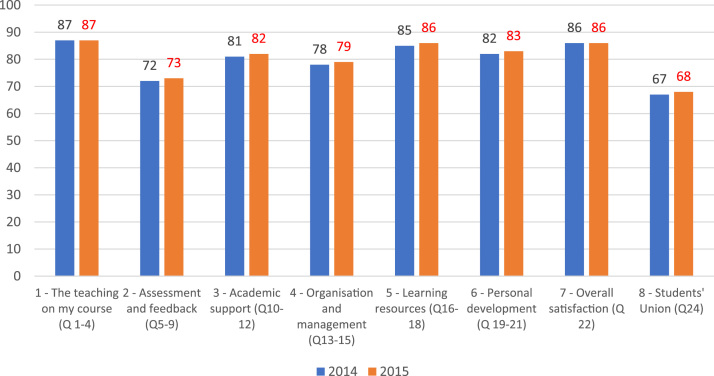
Student satisfaction percentage in 2014 and 2015.

Fig. 1 shows the student satisfaction percentage in 2014 and 2015 based on the NSS Questionnaire survey. Importantly, teaching quality (Q1–4) is the highest satisfaction percentage. But for the assessment and feedback, the students were not satisfied and raised question on the quality of the feedback. In addition, the engagement of student union is not very active for many universities.

To achieve the objective of the study, the study has developed the following hypothesis:

$$\begin{array}{c} Nullhy\;\;pothesis:\;\;\;\;\;\;\;\;\;H_{0}:\mu_{1}-{µ}_{2}=0\\ Alternative\;\;hypothesis:\;\;H_{1}:\mu_{1}-{µ}_{2}\neq 0 \end{array}$$

Where, μ_1_ is the mean number of student satisfaction of Russell Group; μ_2_ is the mean number of student satisfaction of Non-Russell Group

Table 2 reports the Descriptive Statistics of the study. It is found that the mean of the Students satisfaction (Russell Group) is 87 comparing to Students satisfaction (Non-Russell Group) with 85.48. The lower standard deviation of the Russell Group means that the data tend to be closer to the mean. Regarding the Estimation for Difference, we are 95% confident that the confidence interval as: −0.318 ≤ μ_1_–μ_2_ ≤ 3.357 (Table 3).

**Table 2:** Descriptive statistics.

Sample	N	Mean	SD	SE Mean
Students satisfaction (Russell Group)	19	87.00	3.24	0.79
Students satisfaction (Non-Russell Group)	102	85.48	4.35	0.43

**Fig. 2:**
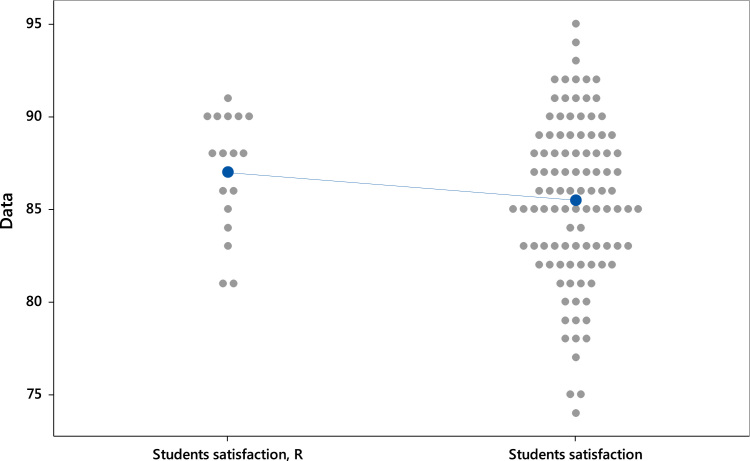
Individual Value Plot of Student Satisfaction. Note: Student Satisfaction, R = Russell Group; Student Satisfaction = Non-Russell Group.

We find that t value is 1.70, *df* is 28 and *p* > .05. This indicates that the null hypothesis is not rejected. Fig. 2 shows the individual Value Plot of Students satisfaction of Russell Group and Non-Russell Group.

**Table 3:** Estimation for difference.

Difference	95% CI for Difference
1.519	(-0.318, 3.357)

Overall, we can conclude that since the null hypothesis is not rejected, the mean of student satisfaction between Russell Group and Non-Russell Group universities are not significantly different.
